# Molecular analyses reveal the occurrence of three new sympatric lineages of velvet worms (Onychophora: Peripatidae) in the eastern Amazon basin

**DOI:** 10.1590/1678-4685-GMB-2016-0037

**Published:** 2017-03-02

**Authors:** Williana T. R. Cunha, Rita C. O. Santos, Juliana Araripe, Iracilda Sampaio, Horacio Schneider, Péricles S. Rêgo

**Affiliations:** 1 Laboratório de Genética e Conservação, Instituto de Estudos Costeiros, Universidade Federal do Pará, Bragança, PA, Brazil; 2 Laboratório de Genética e Biologia Molecular, Instituto de Estudos Costeiros, Universidade Federal do Pará, Bragança, PA, Brazil

**Keywords:** Amazonia, new lineages, Peripatidae, phylogeny, Onychophora

## Abstract

In the present study, we investigated the possible existence of new lineages of peripatids through comparisons between known Neotropical species and specimens obtained from two locations in Pará, a state in eastern Brazilian Amazonia using a molecular approach based on sequences of the mtDNA genes COI, 16Sr RNA, and 18S RNA. The analyses included also sequences of Asian and African taxa for a more systematic understanding of the phylogenetic relationships within the group. The analysis of the COI, 16S rRNA and 18S RNA sequences permitted the identification of three distinct lineages (A, B and C) based on two different phylogenetic approaches (Bayesian methods and ML). The three lineages presented here are completely distinct from all other peripatid taxa so far defined by molecular data. The presence of specimens of three independent onychophoran lineages occurring in sympatry in the Amazon basin was confirmed in all the analyses, providing consistent support for the phylogenies presented in this study. These findings reinforce the importance of the Amazon region in the diversification of Neotropical peripatids, and indicate that onychophoran diversity is much greater than previously thought, given that the number of taxa found at a single site was equivalent to the total number of allopatric species described for the entire region.

Velvet worms are soft-bodied terrestrial invertebrates that inhabit humid forests, where they are typically associated with the soil, decaying trunks, and leaf litter, as well as caves (Vasconcellos *et al*., 2004). These worms are usually confined to stable microhabitats with high levels of humidity, and have limited dispersal capabilities in open environments (New, 1995). Currently, the phylum Onychophora encompasses two widely-distributed but allopatric families: Peripatidae Evans, 1901, found in Central and South America, West Africa, and Southeast Asia; and Peripatopsidae Bouvier, 1905 which is restricted to South Africa, Australasia, and South America (Oliveira *et al*., 2012a).

The family Peripatidae is distributed in 12 genera, with a total of 85 species currently recognized (Oliveira *et al*., 2012a,b, 2013, 2014). Four genera of this family are found in the Amazon basin, where only seven species have been identified so far: *Epiperipatus brasiliensis* (Bouvier, 1889); *Epiperipatus edwardsii* (Blanchard, 1847); *Epiperipatus simoni* (Bouvier, 1899); *Epiperipatus tucupi* (Froehlich, 1968), designated *nomen dubium* by Oliveira *et al*. (2012a); *Macroperipatus geayi* (Bouvier, 1889); *Oroperipatus balzani* (Camerano, 1897); and *Oroperipatus eisenii* (Wheeler, 1898), although Sampaio-Costa *et al*. (2009) have also described a morphospecies of the genus *Peripatus*. While onychophorans are known to occur in the Amazon basin (Read, 1988; Sampaio-Costa *et al*., 2009), the diversity of these terrestrial invertebrates in this region – considered “megadiverse” for many other groups of animals (Mittermeier *et al*., 2003) – is still poorly known, and the last species of velvet worm from the Amazon biome was described more than 50 years ago (Froehlich, 1968).

While there have been recent advances in the description of onychophoran species, more than 70% of the 85 recognized species of Peripatidae are in need of revision. In addition to the new diagnostic characteristics proposed by Oliveira *et al*. (2012b, 2013, 2014), genetic data obtained over the past few decades have been used increasingly to complement morphological analyses, contributing to the identification of cryptic species, the determination of species limits, phylogeny, and the distribution patterns of this invertebrate group (Lacorte *et al*., 2011; Oliveira *et al*., 2011; Murienne *et al*., 2013).

In the present study, we analyzed the sequences of two mitochondrial markers and one nuclear marker in specimens of peripatids collected from fragments of secondary forest in the eastern Brazilian Amazon basin. Based on the results, we investigated the possible existence of new taxa through comparisons with the Neotropical species already analyzed using this approach, for the definition of phylogenetic relationships. The findings also contribute to our knowledge of the natural history of this poorly-known group of Amazonian organisms.

The tissue samples were obtained from 22 specimens collected between 2006 and 2011 from two localities in Pará, a state in eastern Brazilian Amazonia. Specimen collection was authorized by the Brazilian federal environment institute (Sistema de Autorização e Informação em Biodiversidade - SISBIO / Instituto Chico Mendes de Conservação da Biodiversidade – ICMBio; special license 24714-1). The sample localities shown in Figure 1 and listed in Table S1 were: (7) Outeiro Island - OTI (1°14′56″ S, 48°26′57″ W) (*n* = 10 specimens) and (8) Bragança - BRG (1°02′04″ S, 46°45'48″ W) (*n* = 12 specimens), which are separated by a distance of 234 km (Figure 1). The first site (OTI) is a small patch of forest, of approximately 2 km^2^, located within the urban area of Outeiro Island. The habitat is well-shaded, with many decomposing tree trunks on the ground, and is interspersed with plantations of regional fruit species such as “açaí”, *Euterpe oleraceae* Mart. and “cupuaçu”, *Theobroma grandiflorum* (Willd. ex Spreng). The second site (BRG) is a fragment of secondary forest of less than 6 km^2^, with high levels of anthropogenic disturbance, located within the town's urban zone. Specimens were mostly found in rotten trunks, leaf litter, tree roots, and cavities in the ground. The animals were euthanized using the Monge-Nájera and Bernal (1994) protocol and preserved in 70% ethanol, prior to being deposited in the zoological collection of the Bragança *campus* of the Federal University of Pará, Brazil.

**Figure 1:**
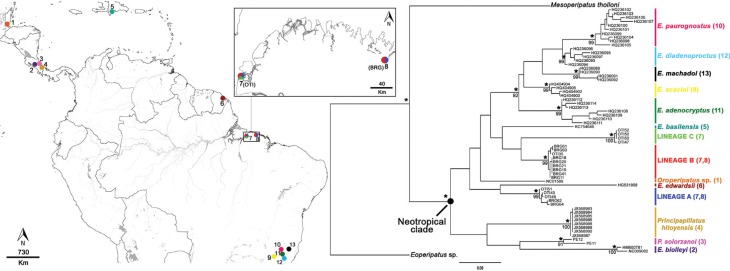
Map showing the geographic distribution of the species of Peripatidae and sites analyzed in the present study. Numbers correspond to locality records listed in Table S1 (Supplementary material). Colours refer to the clades based on the phylogenetic tree. Topology obtained from the ML analyses of mitochondrial COI. Above: asterisks indicated Bayesian posteriors probabilities > 0.9; below: numbers nodes are bootstrap values > 75%. Abbreviations: OTI = Outeiro Island; BRG = municipality of Bragança.

Total DNA was extracted from fragments of the tegument of the specimens using a phenol-chloroform protocol (Sambrook *et al*., 1989). Fragments of two mitochondrial loci, Cytochrome Oxidase I (COI) and the large Ribosomal Subunit (16S rRNA), as well as the nuclear ribosomal gene 18S (18S rRNA) locus, were amplified by polymerase chain reaction (PCR). For COI, the primers were L5584 and H6174 (Oliveira *et al*., 2011), whereas for 16S rRNA, they were L1987 and H2609 (Palumbi *et al*., 1991), and for the nuclear marker 18S rRNA, the primers were 1F and 5R, from Giribet *et al*. (1996).

The nucleotide sequences of the resulting gene fragments were determined in an ABI 3500 automatic sequencer (Applied Biosystems). In addition to the sequences obtained in the present study, COI, 16S rRNA and 18S rRNA sequences were obtained from GenBank for inclusion in the analyses (Table S1), representing 13 species of peripatids, 11 from the Neotropical region, one from tropical Africa and one from Asia, with the latter two being used as the outgroups, based on the results of Murienne *et al*. (2013).

The sequences were aligned in ClustalW (Thompson *et al*., 1994) using the Bioedit v7.0.5 sequence editor (Hall, 1999). The nucleotide composition and divergence rates (p distances) between and within the observed lineages were calculated in MEGA v6.0 (Tamura *et al*., 2013). The JModelTest v2.0.2 program (Guindon and Gascuel 2003; Darriba *et al*., 2012) was used to select the optimal evolutionary model for the phylogenetic analysis of the sequences of both mtDNA and ncDNA regions using the Akaike and Bayesian information criteria (AIC and BIC).

The maximum likelihood (ML) analysis was run in PhyML v3.0 (Guindon and Gascuel, 2003). Support for the groups was evaluated using a bootstrap approach with 1000 replicates. Bootstrap support values of more than 75% were considered to represent a well-defined group. The evolutionary Bayesian inference (BI) models produced by the MrBayes v3.2.0 program (Ronquist and Huelsenbeck, 2003) were selected based on the Bayesian Information Criterion (BIC). The Bayesian Monte Carlo Markov Chain (BMCMC) procedure was based on four chains run simultaneously over 10^7^ generations, with samples being taken every 100 generations. Convergence and effective sample sizes (ESS) were assessed in Tracer v1.6.1 (Rambaut and Drummond, 2007), and the first 100 trees of each run were discarded as burn-in. Bayesian posterior probability values lower than 0.9 were considered to be inconclusive. The trees were visualized in FigTree v.1.4 (Rambaut, 2012).

The best model of nucleotide substitution and the partitioning of the concatenated data set were selected using Partition Finder, v. 1.1.1 (Lanfear *et al*., 2012), based on the Akaike Information Criterion, or AIC (Akaike, 1973). The phylogenetic trees derived from the ML analysis was estimated using RaxML, v. 8.0 (Stamatakis, 2014), with the optimal partitioning for this analysis and the support for each branch node being calculated using a nonparametric bootstrap analysis, with 1000 pseudo-replicates (Felsenstein, 1985), which also provides an estimate of the confidence for the results. The trees were visualized in FigTree v.1.4 (Rambaut, 2012).

It was not possible to sequence all the genes for each of the specimens collected (Table S1). The sequencing of the COI gene produced fragments of 501 base pairs (bps) with 183 variable sites for 18 of the specimens collected for this study. The 16S rRNA was sequenced in 13 specimens, producing fragments of 392 bps, with 97 variable sites. Fragments of the 18S rRNA were amplified in ten specimens, providing 775 bps and 149 variable sites. There was a predominance of the A+T nucleotides in all the markers, which is typical of both onychophorans and most other invertebrates (Trewick, 2000; Jeon *et al*., 2012).

The GTR+ I + G model was selected for the COI sequences for both probabilistic approaches (ML and BI). The K81uf+G, TVM+G, and HKY+G models were selected for the ML analysis based on the concatenated COI, 16S rRNA, and 18S rRNA sequences, respectively. The phylogenetic analyses based on the COI, 16S rRNA and 18S rRNA sequences permitted the identification of three distinct lineages from the Amazon region. The first two lineages, denominated A and B, are made up of specimens from both Outeiro Island and Bragança, while the third, lineage C, was formed exclusively by specimens from Outeiro Island (Figure 1; Figure S1). The topology derived did not provide statistically support for any phylogenetic relationship between the species with sequences available and the lineages identified in the present study. Even though, all the species and lineages represented by more than one specimen were validated with high levels of statistical support, confirming the capacity of the markers to distinguish valid taxa in the onychophorans.

We also confirmed that the three clades identified in the present study do not form a single group. Lineage A appears to be close to *E. edwardsii*, whereas lineages B and C form a sister group to the Caribbean *Peripatus dominicae basilensis*, although in all cases, with reduced statistical support (Figure 1). The data also indicate that the species of the genera *Epiperipatus* and *Peripatus* are non-monophyletic.

The genetic divergence found between the species and the new Neotropical lineages varied from 4.9% to 22.6% when the outgroups were included, and from 4.9% to 20.6% when only the ingroup was considered (Table 1). These values were obtained for the COI marker, which permits the greatest number of comparisons due to the large number of species for which published data are available. The minimum interspecific divergence observed between Amazonian taxa (lineages B and C) was 9.9%, while the maximum was 14.5%, between the A and C lineages. Intraspecific genetic divergence was relatively low, at 0.5% (lineage A), 0.4% (lineage B), and 0.2% (lineage C), similar to that recorded for the species *Principapillatus hitoyensis* (0.2%), and lower than the divergence found in *E. machadoi*, *E. diadenoproctus*, *E. paurognostus* and *E. adenocryptus* (1.4%, 1.1%, 1% and 2%) (Oliveira *et al*., 2011), which indicates low levels of intraspecific variation in the sequences of the specimens analyzed. In the case of the 16S rRNA gene, divergence was between 9% and 12% (including outgroups), with a minimum of 9% between the A and B lineages and 11% between B and C. Intraspecific genetic divergence was 0.4% (lineage A), 0.0% (lineage B), and 0.2% (lineage C). Based on the 18S rRNA gene, the minimum distance was 1.1%, between lineages A and C, while the maximum was 2.2%, between lineages B and C.

**Table 1:** The *p*-distances recorded within and between the lineages and species analyzed in the present study based on sequences of the mitochondrial COI gene.

Lineages / species	within															
		(1)	(2)	(3)	(4)	(5)	(6)	(7)	(8)	(9)	(10)	(11)	(12)	(13)	(14)	(15)
(1) Lineage A	0.005															
(2) Lineage B	0.004	0.115														
(3) Lineage C	0.002	0.145	0.099													
(4) *E. acacioi*	0.013	0.130	0.096	0.128												
(5) *E. paurognostus*	0.031	0.140	0.124	0.165	0.104											
(6) *E. diadenoproctus*	0.013	0.136	0.105	0.140	0.070	0.049										
(7) *E. machadoi*	0.013	0.117	0.092	0.142	0.097	0.103	0.067									
(8) *E. adenocryptus*	0.028	0.134	0.120	0.153	0.080	0.086	0.083	0.110								
(9) *E. edwardsii* a	—	0.127	0.133	0.158	0.138	0.181	0.167	0.155	0.170							
(10) *E. biolleyi*	0.015	0.168	0.141	0.167	0.170	0.206	0.184	0.173	0.201	0.194						
(11) *P. dominicae* a	—	0.127	0.092	0.131	0.099	0.126	0.110	0.118	0.123	0.160	0.149					
(12) *P. solorzanoi*	0.027	0.150	0.126	0.154	0.163	0.175	0.162	0.150	0.169	0.171	0.115	0.142				
(13) *P. hitoyensis*	0.002	0.154	0.141	0.170	0.136	0.164	0.147	0.161	0.136	0.167	0.144	0.139	0.117			
(14) *Oroperipatus sp*.^a^	—	0.098	0.097	0.127	0.101	0.125	0.119	0.128	0.122	0.153	0.142	0.104	0.127	0.139		
(15) *Mesoperipatus tholoni* a	—	0.182	0.159	0.184	0.183	0.198	0.182	0.164	0.196	0.195	0.187	0.175	0.172	0.185	0.167	
(16) *Eoperipatus sp*.^a^	—	0.162	0.195	0.204	0.209	0.226	0.206	0.183	0.215	0.221	0.200	0.199	0.202	0.201	0.182	0.203

a species represented by a single specimen.

The comparative analysis of the specimens, together with the other peripatid taxa for which molecular data are available, indicates that they represent three completely distinct lineages. The phylogenies based on the mitochondrial and nuclear sequences, and the marked divergence found between the lineages identified in the analyses (9-13%) indicate the existence of distinct species (Table 1), considering the phylogenetic species concept (*sensu* Mishler and Theriot, 2000). In fact, a genetic distance of only 4.4% was considered diagnostic of the species-level differentiation of the allopatric peripatids *E*. *adenocryptus* and *E*. *paurognostus* (Oliveira *et al*., 2011).

As the type localities of three onychophoran species, *M. geayi*, *E. brasiliensis*, and *E. tucupi* (Sampaio-Costa *et al*., 2009), are located in the eastern Amazon basin, it seems likely that at least one of the lineages identified in the present study (which need to be described formally) may correspond to one of these species. However, as the type-specimen for these species are by now too degraded to provide material for genetic analysis and no other specimens are available from the type localities, it is impossible to provide a direct comparison using molecular tools. In addition, there is no information on the exact geographical location of the type localities, which impedes the collection of new specimens. Clearly, it will be necessary to examine the specimens analyzed in the present study very meticulously for the identification of diagnostic traits in order to confirm their potential species status and avoid synonymy.

The present analysis of mitochondrial and nuclear markers did not provide a well-resolved arrangement (Figure 1; Figure S1). The Amazonian lineages do not form a single clade when analyzed in comparison with the other species for which data are available on the same molecular parameters. This finding contrasts with the situation observed in five allopatric species of *Epiperipatus* from the state of Minas Gerais, Brazil (Oliveira *et al*., 2011). While the latter are separated by distances of between 11 km and 155 km, they are nevertheless phylogenetically closely related, forming a single group with a common ancestor.

The sympatric occurrence of these phylogenetically distinct lineages also indicates that this group of animals has undergone distinct spatial-temporal differentiation processes, which have molded species ranges and their diversity in this biome, as observed in other groups of organisms, reflecting the complex zoogeographic and cladogenetic processes that are typical of the Amazon biome. While these animals are restricted to humid habitats, the different lineages are probably adapted to distinct ecological conditions. In the Blue Mountains of Australia, for example, the sympatric species *Cephalophovea tomahmontis* Ruhberg, Tait, Briscoe and Storch, 1988 and *Euperipatoides leuckartii* (Sänger, 1871) present quite distinct life history strategies (Leishman and Eldridge, 1990). A high level of interspecific variation (9.22%) has been observed in sympatric peripatopsids, between the *Peripatopsis moseleyi* (Wood-Mason, 1879) and *Peripatopsis balfouri* (Sedgwick, 1885) species complexes (Daniels and Ruhberg, 2010; Daniels *et al*., 2013).

An interesting aspect of the results of our study is the occurrence in Bragança of two of the three lineages found on Outeiro Island, 234 km to the west (Figure 1). A probable scenario is that the lineages were more amply distributed prior to the formation of the island, with the present-day distribution attesting to the ancient connectivity of these environments. Given this situation, the restriction of lineage C to Outeiro Island may reflect a sampling effect rather than the presence of an endemic taxon on the island, which was not identified as a vicariant factor in the establishment of any of the lineages. It would thus be important to expand the number of points sampled on the mainland in order to confirm the more ample distribution of all three lineages.

The small number of available specimens and sample localities are insufficient for a more conclusive analysis of geographic limits or distribution patterns of the three lineages, although they do confirm their occurrence. These findings reinforce the importance of the Amazon region in the diversification of the Neotropical peripatids, and indicate that onychophoran diversity is much greater than previously thought, given that the number of taxa found at a single site was equivalent to the total number of allopatric species described for the entire region (Sampaio-Costa *et al*., 2009). Similarly, understanding how the velvet worms colonized the region, and which barriers contributed to their diversification, may provide important insights into speciation patterns in the Amazon basin, given that onychophorans have low vagility and are sensitive to environmental impacts. These characteristics may favor the isolation of these organisms, making them an appropriate model for the analysis of biogeographic patterns (Murienne *et al*., 2013).

## Supplementary material

The following online material is available for this article:

Figure S1 - Maximum-likelihood tree for three genes (COI, 16S, 18S).

Table S1 - Specimen information.

## References

[B1] Akaike H, Petrov BN, Csaki F (1973). Information theory and an extension of the maximum
likelihood principle. Second International Symposium on Information Theory.

[B2] Daniels SR, Ruhberg H (2010). Molecular and morphological variation in a South African velvet worm
*Peripatopsis moseleyi* (Onychophora, Peripatopsidae): Evidence
for cryptic speciation. J Zool.

[B3] Daniels SR, McDonald DN, Picker MD (2013). Evolutionary insight into the *Peripatopsis balfouri*
sensu lato species complex (Onychophora: Peripatopsidae) reveals novel lineages
and zoogeographic patterning. Zool Scr.

[B4] Darriba D, Taboada GL, Doallo R, Posada D (2012). jModelTest 2: More models, new heuristics and parallel
computing. Nat Methods.

[B5] Felsenstein J (1985). Confidence limits on phylogenies: An approach using the
bootstrap. Evolution.

[B6] Froehlich CG (1968). On some Brazilian onychophores. Beitr Neotrop Fauna.

[B7] Giribet G, Carranza S, Baguñà J, Riutort M, Ribera C (1996). First molecular evidence for the existence of a Tardigrada +
Arthropoda clade. Mol Biol Evol.

[B8] Guindon S, Gascuel O (2003). A simple, fast, and accurate algorithm to estimate large phylogenies
by maximum likelihood. Syst Biol.

[B9] Hall TA (1999). BIOEDIT: A user-friendly biological sequence alignment editor and
analysis program for Windows 95/98/NT. Nucleic Acids Symp.

[B10] Jeon MJ, Song JH, Ahn KJ (2012). Molecular phylogeny of the marine littoral genus
*Cafius* (Coleoptera: Staphylinidae: Staphylininae) and
implications for classification. Zool Scr.

[B11] Lacorte GA, Oliveira IS, Fonseca CG (2011). Phylogenetic relationships among the *Epiperipatus*
lineages (Onychophora: Peripatidae) from the Minas Gerais State,
Brazil. Zootaxa.

[B12] Lanfear R, Calcott B, Ho SYW, Guindon S (2012). PartitionFinder: Combined selection of partitioning schemes and
substitution models for phylogenetic analyses. Mol Biol Evol.

[B13] Leishman MR, Eldridge DB (1990). Life history characteristics of two sympatric onychophoran species
from the blue mountains, New South Wales. Proc Linn Soc New South Wales.

[B14] Mishler BD, Theriot EC, Wheeler QD, Meier R (2000). The phylogenetic species concept (*sensu*
Mishler and Theriot): Monophyly, apomorphy, and phylogenetic species
concepts. Species Concepts and Phylogenetic Theory: A Debate.

[B15] Mittermeier RA, Mittermeier CG, Brooks TM, Pilgrim JD, Konstant WR, da Fonseca GAB, Kormos C (2003). Wilderness and biodiversity conservation. Proc Natl Acad Sci U S A.

[B16] Monge-Nájera J, Bernal MB (1994). Morphological and physiological characteristics of two species of
*Epiperipatus* from Costa Rica (Onychophora:
Peripatidae). Rev Biol Trop.

[B17] Murienne J, Daniels SR, Buckley TR, Mayer G, Giribet G (2013). A living fossil tale of Pangaean biogeography. Proc R Soc Lond B Biol Sci.

[B18] New TR (1995). Onychophora in invertebrate conservation: Priorities, practice and
prospects. Zool J Linn Soc.

[B19] Oliveira IS, Lacorte GA, Fonseca CG, Wieloch AH, Mayer G (2011). Cryptic speciation in Brazilian *Epiperipatus*
(Onychophora: Peripatidae) reveals an underestimated diversity among the peripatid
velvet worms. PLoS One.

[B20] Oliveira IS, Read VMSJ, Mayer G (2012a). A world checklist of Onychophora (velvet worms), with notes on
nomenclature and status of names. Zookeys.

[B21] Oliveira IS, Franke FA, Hering L, Schaffer S, Rowell DM, Weck-Heimann A, Monge-Nájera J, Morera-Brenes B, Mayer G (2012b). Unexplored character diversity in Onychophora (velvet worms): A
comparative study of three peripatid species. PLoS One.

[B22] Oliveira IS, Schaffer S, Kvartalnov PV, Galoyan EA, Palko IV, Weck-Heimann A, Geissler P, Ruhberg H, Mayer G (2013). A new species of *Eoperipatus* (Onychophora) from
Vietnam reveals novel morphological characters for the South-East Asian
Peripatidae. Zool Anz.

[B23] Oliveira IS, Lacorte GA, Weck-Heimann A, Cordeiro LM, Wieloch AH, Mayer G (2014). A new and critically endangered species and genus of Onychophora
(Peripatidae) from the Brazilian savannah - A vulnerable biodiversity
hotspot. Syst Biodivers.

[B24] Palumbi S, Martin A, Romano S, McMillian WO, Stice L, Grabowiski G (1991). The Simple Fool's Guide to PCR.

[B25] Read VMSJ (1988). The application of scanning electron microscopy to the systematics of
the Neotropical Peripatidae (Onychophora). Zool J Linn Soc.

[B26] Ronquist F, Huelsenbeck JP (2003). Mrbayes 3: Bayesian phylogenetic inference under mixed
models. Bioinformatics.

[B27] Sambrook J, Fritsch E, Maniatis T (1989). Molecular Cloning: A Laboratory Manual.

[B28] Sampaio-Costa C, Chagas-Junior A, Baptista RLC (2009). Brazilian species of Onychophora with notes on their taxonomy and
distribution. Zoologia.

[B29] Stamatakis A (2014). RAxML version 8: A tool for phylogenetic analysis and post-analysis of
large phylogenies. Bioinformatics.

[B30] Tamura K, Stecher G, Peterson D, Filipski A, Kumar S (2013). MEGA6: Molecular Evolutionary Genetics Analysis version
6.0. Mol Biol Evol.

[B31] Thompson JD, Higgins DG, Gibson TJ (1994). Clustal W: Improving the sensitivity of progressive multiple sequence
alignment through sequence weighting, position-specific gap penalties and weight
matrix choice. Nucleic Acids Res.

[B32] Trewick SA (2000). Mitochondrial DNA sequences support allozyme evidence for cryptic
radiation of new zealand Peripatoides (Onychophora). Mol Ecol.

[B33] Vasconcellos A, Almeida WO, Eloy ECC, Pôrto KC, Cabral JJP, Tabarelli M (2004). Onychophora de florestas úmidas do complexo da mata
atlântica do nordeste brasileiro e sua importância para conservação e estudos
sistemáticos. Brejos de Altitude: História Natural, Ecologia e Conservação.

