# Supplementary material for: Molecular analyses reveal the occurrence of three new sympatric lineages of velvet worms (Onychophora: Peripatidae) in the eastern Amazon basin
Source: Genet Mol Biol. 2017 Mar 2;40(1):147–52. doi: 10.1590/1678-4685-GMB-2016-0037 (PMC5409768; doi:10.1590/1678-4685-GMB-2016-0037)
Supplement: Supplementary file 1 [file 1415-4757-gmb-1678-4685-GMB-2016-0037-Suppl02.pdf]

Table S1 − Specimen numbers/lineage, species, localities numbered (N) as in the map (Figure 1), GenBank access codes used in our analyses and references.

			GenBank accession number			
**Sample/Lineage**	**Locality**	**N**	**COI**	**16S rRNA**	**18S rRNA**	**Reference**
OTI 035/B	Brazil: Pará: Ilha de Outeiro	7	KX394477	—	—	Present study
OTI 043/A	Brazil: Pará: Ilha de Outeiro	7	KX394478	KX394458	KX394472	Present study
OTI 047/C	Brazil: Pará: Ilha de Outeiro	7	KX394479	KX394460	KX394474	Present study
OTI 048/A	Brazil: Pará: Ilha de Outeiro	7	—	—	KX394473	Present study
OIT 049/A	Brazil: Pará: Ilha de Outeiro	7	KX394480	—	—	Present study
OIT 051/A	Brazil: Pará: Ilha de Outeiro	7	KX394481	KX394455	KX394471	Present study
OTI 050/C	Brazil: Pará: Ilha de Outeiro	7	KX394483	KX394459	KX394476	Present study
OTI 052/C	Brazil: Pará: Ilha de Outeiro	7	KX394482	KX394456	—	Present study
OTI 053/C	Brazil: Pará: Ilha de Outeiro	7	KX394484	—	KX394475	Present study
OTI 054/B	Brazil: Pará: Ilha de Outeiro	7	—	KX394457	—	Present study
BRG 064/A	Brazil: Pará: Bragança	8	KX394485	KX394461	KX394470	Present study
BRG 011/B	Brazil: Pará: Bragança	8	KX394486	—	—	Present study
BRG 014/B	Brazil: Pará: Bragança	8	—	KX394462	—	Present study
BRG 015/B	Brazil: Pará: Bragança	8	KX394487	—	—	Present study
BRG 018/B	Brazil: Pará: Bragança	8	KX394488	KX394463	—	Present study
BRG 020/B	Brazil: Pará: Bragança	8	KX394489	KX394464	—	Present study
BRG 021/B	Brazil: Pará: Bragança	8	KX394490	—	—	Present study
BRG 033/B	Brazil: Pará: Bragança	8	—	—	KX394469	Present study
BRG 041/B	Brazil: Pará: Bragança	8	KX394491	KX394465	KX394468	Present study
BRG 061/B	Brazil: Pará: Bragança	8	KX394492	KX394466	KX394469	Present study
BRG 062/A	Brazil: Pará: Bragança	8	KX394493	—	—	Present study
BRG 063/B	Brazil: Pará: Bragança	8	KX394494	KX394467	—	Present study

Species	Locality	N	GenBank accession number COI	16S rRNA	18S rRNA	Reference
Epiperipatus edwardsii (Blanchard, 1847)	French Guiana: Cayenne	6	HG531958	HG531962	HG531959	Murienne *et al.*, (2013)
Epiperipatus biolleyi (Bouvier, 1902)	Costa Rica: San José	2	NC009082	NC009082	AF370782	Podsiadlowski *et al.*, (2008)[a] Giribet *et al.*, (2001)[b]
	Costa Rica: Cascajal de Coronado		HM600781	HM600781	—	Rota-Stabelli *et al.*, (2010)[c]
Epiperipatus acacioi (Marcus & Marcus, 1955)	Brazil: Minas Gerais	9	HQ404902-05	—	—	Lacorte *et al.* (2011)
Epiperipatus machadoi (Oliveira & Wieloch, 2005)	Brazil: Minas Gerais	13	HQ236089-92	—	—	Lacorte *et al.* (2011)
Epiperipatus adenocryptus Oliveira *et al.*, 2011	Brazil: Minas Gerais	11	HQ236108-14	—	—	Oliveira *et al.* (2011)
Epiperipatus diadenoproctus Oliveira *et al.*, 2011	Brazil: Minas Gerais	12	HQ236093-97	—	—	Oliveira *et al.* (2011)
Epiperipatus paurognostus Oliveira *et al.*, 2011	Brazil: Minas Gerais	10	HQ236098-6107	—	—	Oliveira *et al.* (2011)
Peripatus dominicae basilensis Brues,1935	Dominican Republic	5	KC754646	—	KC754479	Oliveira *et al.* (2014)
Peripatus solorzanoi Morera-Brenes & Monge-Nájera, 2010	Costa Rica: Limón	3	PE11-12	—	—	Morera-Brenes and Monge-Nájera (2010)[d]
Principapillatus hitoyensis Oliveira *et al.*, 2012	Costa Rica: Limón	4	JX568983-90	—	—	Oliveira *et al.* (2012b)
	Costa Rica: Limón	4	—	KC754525	KC754575	Murienne *et al.* (2013)
Oroperipatus sp.	Costa Rica: Belize	1	NC01589	NC01589	—	Segovia *et al.* (2011)[e]
Eoperipatus sp.	Thailand: Chanthaburi Mountain	—	JX569005	—	—	Oliveira *et al.* (2012b)
	Malaysia	—	—	KC754519	—	Muriene *et al.* (2013)
Mesoperipatus tholloni (Bouvier, 1898)	Gabon	—	KC754645	KC754528	KC754576	Murienne *et al.* (2013)

[a]Podsiadlowski L, Braband A and Mayer G (2008) The complete mitochondrial genome of the onychophoran *Epiperipatus biolleyi* reveals a unique transfer RNA set and provides further support for the Ecdysozoa hypotesis. Mol Biol Evol 25:42–51.

[b]Giribet G, Edgecombe GD and Wheeler WC (2001) Arthropod phylogeny based on eight molecular loci and morphology. Nature 413:157–161.

[c]Rota-Stabelli O, Kayal E, Gleeson D, Daub J, Boore JL, Telford MJ, Pisani D, Blaxter M and Lavrov DV (2010) Ecdysozoan mitogenomics: evidence for a common origin of the legged invertebrates, the Panarthropoda. Genome Biol Evol 2:425–440.

[d]Morera-Brenes B and Monge-Nájera J (2010) A new giant species of placented worm and the mechanism by which onychophorans weave their nets (Onychophora: Peripatidae). Rev Biol Trop 58:1127–1142.

[e]Segovia R, Pett W, Trewick S and Lavrov DV (2011) Extensive and evolutionarily persistent mitochondrial tRNA editing in velvet worms (phylum Onychophora). Mol Biol Evol 28:2873–2881.
