# Supplementary material for: Molecular analyses reveal the occurrence of three new sympatric lineages of velvet worms (Onychophora: Peripatidae) in the eastern Amazon basin
Source: Genet Mol Biol. 2017 Mar 2;40(1):147–52. doi: 10.1590/1678-4685-GMB-2016-0037 (PMC5409768; doi:10.1590/1678-4685-GMB-2016-0037)
Supplement: Supplementary file 2 [file 1415-4757-gmb-1678-4685-GMB-2016-0037-Suppl01.pdf]

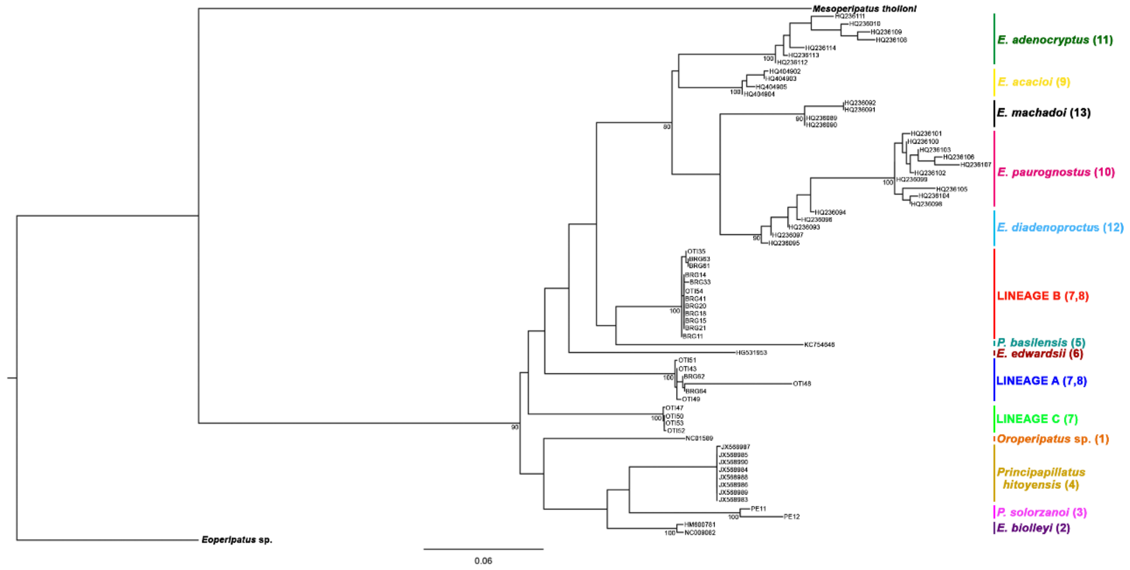

Figure S1- Maximum-likelihood tree inferred of the concatenated dataset for three genes (COI, 16S, 18S). Below: numbers on nodes are bootstrap values >75%. Abbreviations: OTI = Outeiro Island; BRG = municipality of Bragança. Colours refer to the clades based on the map.
